# The Universal and Primary Prevention of Foetal Alcohol Spectrum Disorders (FASD): A Systematic Review

**DOI:** 10.1007/s10935-021-00658-9

**Published:** 2022-02-05

**Authors:** Britta Jacobsen, Christina Lindemann, Rainer Petzina, Uwe Verthein

**Affiliations:** 1 Department of Psychiatry, Centre for Interdisciplinary Addiction Research of Hamburg University (ZIS), University Medical Center Hamburg-Eppendorf (UKE), Hamburg, Germany; 2 Medical School Hamburg (MSH), University of Applied Sciences and Medical University, Hamburg, Germany

**Keywords:** Foetal alcohol spectrum disorders (FASD), Primary prevention, Universal prevention, Pregnancy, Alcohol exposed risk groups, Alcohol consumption

## Abstract

Foetal alcohol spectrum disorder (FASD) comprises multiple neurodevelopmental disorders caused by alcohol consumption during pregnancy. With a global prevalence rate of 7.7 per 1000 population, FASD is a leading cause of prenatal developmental disorders. The extent of physical, mental, and social consequences for individuals with FASD can be vast and negatively affect their social environment, daily life, school, relationships, and work. As treatment for FASD is labour- and cost-intensive, with no cure available, prevention is key in reducing FASD prevalence rates. As most systematic reviews conducted so far have focused on specific FASD risk groups, we investigated the effectiveness of universal FASD prevention and primary preventive strategies. We identified a total of 567 potentially pertinent records through PubMed, Cochrane Library, EBSCO, PubPsych, and DAHTA published from 2010 to May 2020, of which 10 studies were included in this systematic review. Results showed a substantial heterogeneity in the studies’ quality, although all preventive measures, except one, proved effective in both increasing knowledge and awareness of FASD, as well as decreasing the risk of an alcohol exposed pregnancy. Limiting factors such as small sample sizes and a lack of behavioural change testing require further studies to support existing evidence for FASD prevention and its implementation, as well as detecting the best course of action for FASD prevention when creating and implementing prevention and intervention approaches.

## Introduction

Foetal alcohol spectrum disorder (FASD) is an umbrella term for preventable foetal developmental disorders (Symons et al., 2018) that cannot be cured. The estimated prevalence of an FASD diagnosis among minors in Europe is the highest in the world, with 19.8 per 1000 population, whereas the global prevalence is 7.7 per 1000 (Lange et al., 2017).

The effects of alcohol consumption during pregnancy are manifold and can have serious consequences for both the child and its family. The extent of abnormalities linked to antenatal alcohol consumption can range from (birth) deformities, organic dysfunctions, disabilities, and behavioural problems to mental disorders and is related to the amount of alcohol consumed and the stage of gestational development during the period of consumption (Coriale et al., 2013; Denny et al., 2017; Lohaus & Vierhaus, 2015; Ornoy & Ergaz, 2010; Wilhoit et al., 2017). Additionally, individuals born with FASD can experience problems in emotional affect regulation and are at an increased risk for suicide (Temple et al., 2019) and for developing mental health problems like conduct disorders, substance abuse (Popova et al., 2018), and disorders relating to hyperactivity and attention deficits (Mukherjee et al., 2006). The comorbid occurrence of mental disorders can also be due to childhood traumatic experiences, including adverse childhood experiences such as neglect and abuse, or an indirect consequence of typical dysfunctional behaviour and cognition in FASD individuals, and may lead to further neurodevelopmental changes and impairments (Price et al., 2017; Wilhoit et al., 2017). Common FASD impairments can lead to academic failure and impede the affected individual in obtaining and maintaining employment, as well as living independently (Popova et al., 2018). Families’ and caregivers’ quality of life, specifically their involvement in society and stress perception, can also be negatively affected by FASD (Reid & Moritz, 2019). Because the degree of impact is directly associated with the level of the FASD impairment (Reid & Moritz, 2019), severe cases of FASD may have a strong effect on peers and the environment, as they require more care and are cost intensive.

Aside from higher infant mortality and increased overall mortality, pregnant women who consume alcohol are at risk for complications during pregnancy, which can lead to their death or to miscarriages and stillbirths (Popova et al., 2016).

Although FASD symptoms can be treated, the heterogeneous etiopathology and thus wide range of possible impairments increase the difficulties in creating an overarching, homogenous approach for clinical treatment (Coriale et al., 2013). Additionally, research has shown that individuals affected by FASD are oftentimes either misdiagnosed or not diagnosed at all (Denny et al., 2017; Fröschl et al., 2013). Indeed, it has been estimated that approximately only 10% of all children affected by FASD are diagnosed correctly (Fröschl et al., 2013).

Women can prevent FASD by abstaining from alcohol during pregnancy, as well as utilising effective contraceptive behaviour to prevent an unwanted pregnancy (Denny et al., 2017). Given that there is no safe amount of prenatal alcohol consumption (PAC), alcohol abstinence during pregnancy is always advised (Denny et al., 2017; France et al., 2013).

Interventions to prevent FASD are multifaceted. Aside from primary or secondary preventions and interventions amongst women who are pregnant, educating indicated risk groups is vital for FASD prevention. The main target group for FASD prevention is the alcohol-exposed pregnancy risk group (AEPRG), which consists of women of childbearing age (15–49 years old) who consume alcohol regularly, are sexually active, and show ineffective or non-existent contraceptive behaviour (Balachova et al., 2015; Johnson et al., 2010). More specific risk groups are described in the literature, such as indigenous women (Symons et al., 2018), substance abusing women (Bailey & Sokol, 2011), and mentally ill women (Theunissen et al., 2011). Additionally, women who have already conceived a child with FASD are more likely to give birth to more children with FASD when they continue drinking (Montag, 2016), with the risk rising with each subsequent alcohol-exposed pregnancy (Abel, 1988). Researchers have reported different, sometimes contradictory, findings concerning specific risk groups, such as that women of both a high (Landgraf & Heinen, 2016b) and low (Gupta, Gupta, & Shirasaka, 2016; May & Gossage, 2011) socioeconomic status are more likely to bear a child with FASD, findings that may be due to highly selective population samples (Landgraf & Heinen, 2016b). Given the ambiguity of indicated risk groups, as well as a lack of research regarding the effectiveness of universal prevention programs, a review of primary and universal interventions and preventions amongst AEPRGs is needed.

The aim of this systematic review is to compile studies on primary and universal FASD prevention strategies and assess their effectiveness in preventing FASD.

## Method

Our review was limited to peer-reviewed papers reporting studies that were conducted in Europe, North America, and Australia and published in English and German from 2010 to May 2020. Another inclusion criteria consisted of universal, structural, and primary prevention and intervention strategies. Those on a structural or environmental level are defined as being aimed at structures that may constrain or reduce the development of FASD, such as higher taxation on alcoholic goods (Fröschl et al., 2013), those on a primary level that support prevention efforts before FASD can occur, and those on a universal level addressing entire populations. Studies that primarily focussed on specific risk groups or pregnant women were excluded.

The databanks searched included PubMed, Cochrane Library, PubPsych, DAHTA databank, and EBSCO. We adapted our search terms from previous systematic reviews on FASD prevention by Symons et al. (2018) and by Ospina et al. (2015). Our search terms were as follows:

(pregnan OR fetus OR fetal OR prenatal OR in utero OR intrauterine OR maternal exposure) AND (fetal alcohol syndrome OR fetal alcohol OR fasd OR fae OR arbd OR arnd OR prenatal alcohol exposure OR pae OR alcohol exposed pregnancy OR aep) AND alcohol AND (reduc OR prevent OR preventive health services OR primary prevention OR primary intervention OR universal prevention OR product labeling OR campaign OR counselling OR harm reduction OR government programs OR community health services OR health promotion OR health education OR social control policies)

The studies included here were assessed by the quality rating system developed by Moncrieff et al. (2001). It consists of 23 items that measure areas such as bias, comparability, transparency, and sample quality on a scale of 0 to 2, with 2 being the highest rating. If any given item was not applicable, it was excluded. Percentages of the results were then calculated by comparing the number of points achieved with the number of points that could have been achieved for each individual study as to be able to compare the results among all studies.

## Results

**Fig. 1:**
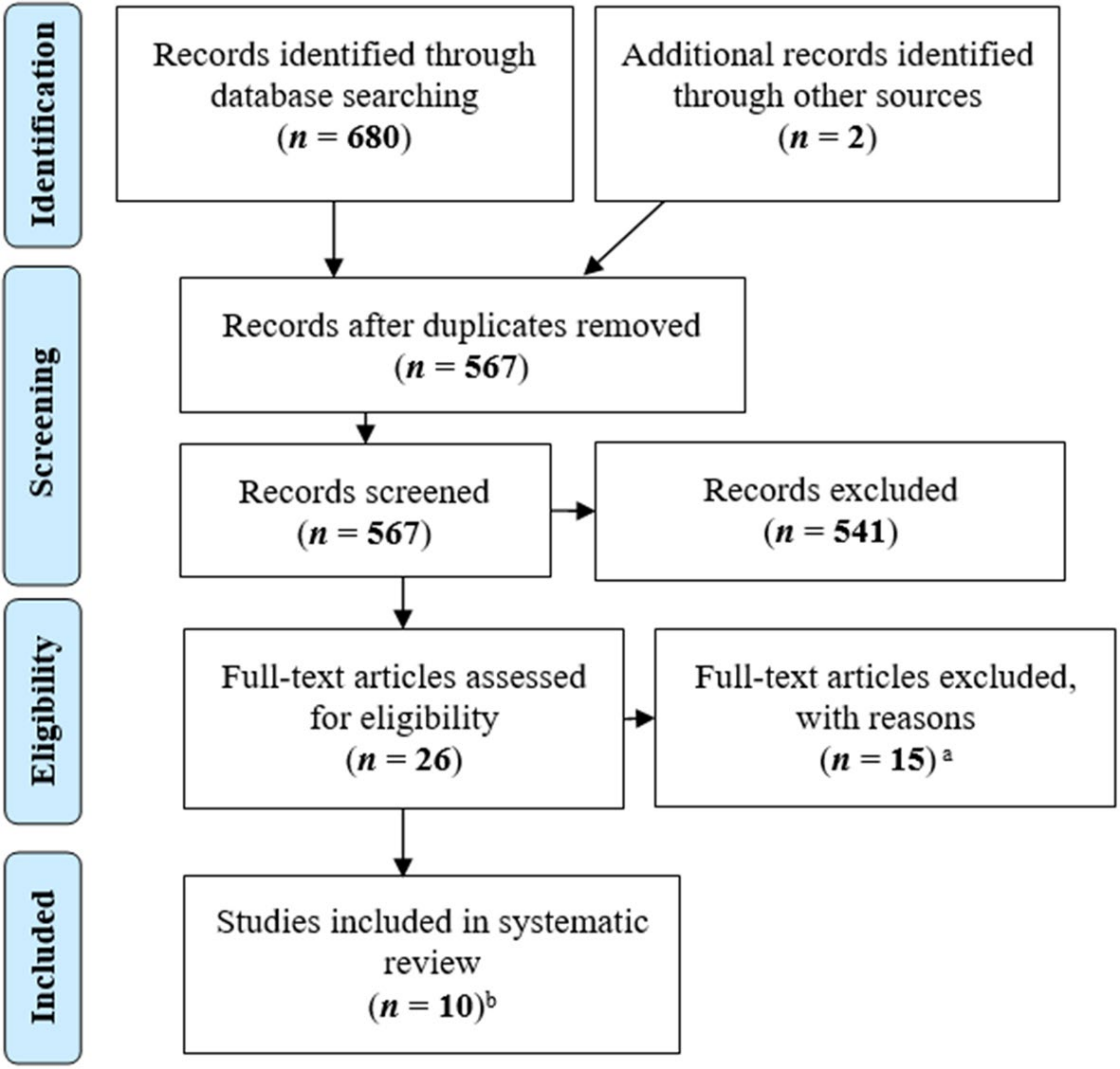
Flow diagram of systematic review process. *Note.* Prisma 2009 Flow Diagram from Moher et al. (2009). ^a^Exclusion reasons (multiple): efficacy of preventions not measured (*n* = 7), guideline (*n* = 1), exclusion criteria (*n* = 5), focus not on FASD (*n* = 4). ^b^Two full-text articles presented the same study and were combined for assessment

After removing duplicates, we identified a total of 567 records. Based on the inclusion criteria, we included 11 publications in the further analysis. We combined two publications that presented the same study (France et al., 2013, 2014); thus, we included 10 studies in the systematic review. An overview can be seen in the following Fig. 1.

We found a huge variety in the study designs, including four randomised controlled trials (RCTs), a secondary data analysis, an observational comparative study, a case control study, a formative evaluation, a non-randomised pre-post intervention study, and an experimental randomised controlled trial. Seven out the 10 studies had follow-ups (Bazzo et al., 2015; Caley et al., 2010; Driscoll et al., 2018; Ingersoll et al., 2013, 2018; Tenkku et al., 2011; Wilton et al., 2013), while Cil (Cil, 2017) compared administrative data over multiple years. Only one study investigated FASD incidence rates (Cil, 2017).

Most studies were conducted in the USA (*n* = 8), while the other two took place in Italy (Bazzo et al., 2015) and Australia (France et al., 2013, 2014). Four studies consisted of samples of AEPRG (Ingersoll et al., 2013, 2018; Tenkku et al., 2011; Wilton et al., 2013), four further studies of women in general (Cil, 2017; Driscoll et al., 2018; France et al., 2013, 2014; Yu et al., 2010), following one including both healthcare professionals and alcohol exposed pregnancy (AEP) risk groups (Bazzo et al., 2015) and one of solely healthcare professionals (Caley et al., 2010). Four studies only included non-pregnant women (Ingersoll et al., 2013, 2018; Tenkku et al., 2011; Wilton et al., 2013), one included only pregnant women (Bazzo et al., 2015), and three addressed both pregnant and non-pregnant women (Driscoll et al., 2018; France et al., 2013, 2014; Yu et al., 2010).

The intervention and prevention types employed in the studies were six on a universal level (including one on a structural level) and four primary preventions for the specific target group of AEPRG. The structural level intervention in the study by Cil (2017) analysed the effect of alcohol warning signs or labels (AWS) placed on alcoholic beverages on birth outcomes on a statewide level. Interventions on a universal level implemented message framing effects, i.e., the effects and influence of the presentation of information on our decision making, (France et al., 2013, 2014; Yu et al., 2010), awareness and educational posters in women’s restrooms (Driscoll et al., 2018), multilevel FASD awareness programs (Bazzo et al., 2015), and educational workshops on screenings and interventions for healthcare professionals (Caley et al., 2010). The interventions for the specific target group AEPRG included web-based and/or mail-based interventions (Ingersoll et al., 2018; Tenkku et al., 2011), video, brochure, or motivational interviewing (Ingersoll et al., 2013), and telephone or in-person interventions (Wilton et al., 2013). The outcomes assessed were drinking and contraceptive behaviour, FASD and prenatal alcohol consumption awareness, knowledge, beliefs and opinions, self-efficacy, symptoms of mental illness and drug use, as well as intervention implementation behaviour.

Self-reports (Bazzo et al., 2015; Driscoll et al., 2018) and Timeline Followbacks (TLFB; Sobell & Sobell, 1992) (Ingersoll et al., 2013, 2018; Tenkku et al., 2011; Wilton et al., 2018) were used as the method for measuring alcohol consumption. The TLFB was also used to assess contraceptive behaviour, although not every study that measured this outcome used a TLFB. In addition to the TLFB, Tenkku et al. (2011) and France et al., (2013, 2014) also implemented the Alcohol Use Disorders Identification Test (AUDIT), while Ingersoll et al. (2013) used the MINI Module J to screen for alcohol use disorders.

The study that received the highest quality rating was Ingersoll et al. (2018) with 90.4%, followed by Cil (2017) with 90.0%, and Wilton et al. (2013), Ingersoll et al. (2013), and Driscoll et al. (2018), with 87.5%, 81.8%, and 78.6%, respectively. The lowest rated study was by Caley et al. (2010) with 52.9%. A brief description of the studies we included in this review can be found in Table 1.

**Table 1:** Characteristics of the included studies

Author (year) Location Study Design and Prevention Type	Participant Details	Intervention Details	Outcome Measures	Results	Quality Rating^a^
Bazzo et al. (2015) Italy Observational Comparative Study design Universal	4 groups; *n* = 101 THCP (41% midwives, 26% gynaecologists, 34% nurses; mean age of 44 years), *n* = 88 VHCP (44% midwives, 47% physicians, 9% nurses; mean age of 35 years), *n* = 127 TPW (mean age of 31.8 years) and *n* = 123 VPW (mean age of 33.7 years)	Multilevel FASD awareness program FASD action research experience for and training of HCP; an FASD communication campaign “*Mamma Beve Bimbo Beve*” for the childbearing-aged population, including advertising messages and print materials	Awareness of the risks of prenatal alcohol exposure Two surveys targeted at HCP and two surveys targeted at pregnant women that were semi-structured self-report questionnaires; Testing of recognition of issue of PAC, rational approach to alcohol, knowledge of alcohol and pregnancy, advice on PAC, opinion on PAC, source of knowledge	Comparison of one and four years after launch of campaign in Treviso: campaign had long term positive effects on HCP, while no difference could be found on cognitive patterns concerning PAC between TPW and VPW; HCP methods probably not effective in changing women’s attitudes and behaviours	26/42 (15,16)^b^ or 61.9%
Caley et al. (2010) USA Case–Control Study Universal	1 group; *n* = 61 health and human service professionals from upstate New York (inclusion criteria: potential to counsel on FASD prevention)	FASD workshop based on constructivist learning theory (3 h)	FASD intervention implementation One-group post-test-only questionnaire on which FASD interventions from a possible 60 had been implemented and how often; follow-up after workshop (4 months)	Increase in FASD interventions 61% (*n* = 37) undertook FASD interventions since the workshop; 226 interventions in 74 different worksites (hospitals = 20%, community outreach = 12%, physician offices = 9%, schools = 8%, etc.) were conducted; frequent methods were posters, pamphlets, and educational material (8.6%) and adding information in prenatal packets (7.7%)	18/34 (3,5,6,13, 14,15)^b^ or 52.9%
Cil (2017) USA Secondary Data Analysis Structural	Women, new-borns	Alcohol warning signs or labels (AWS) on alcoholic products	PAC, PBD and birth outcomes NVS, birth outcome and BRFSS data was compared	AWS laws associated with decrease in odds of PAC (11% decrease) and PBD (75% decrease); No significant difference in AWS laws on FASD birth outcome; Change in PAC largest for first-time mothers and women over 30 years old	27/30 (2,5,6,8,11,13,15,16)^b^ or 90.0%
Driscoll et al. (2018) USA Formative Evaluation Study Universal	2 groups; *n* = 2132 women at baseline (137 pregnant women); *n* = 1182 women at baseline and follow-up (71 pregnant women at follow-up); All participants were 21 years old or older	Awareness and educational posters FASD health communication messages placed on pregnancy test dispenser or as a poster in women’s restrooms in places serving alcohol;	Effectiveness, acceptability, and feasibility of FASD prevention messages in women’s restrooms (including knowledge and beliefs) Questionnaire (where message was placed, pregnancy status/ history, drinking behaviour, FASD knowledge); follow-up 6 months later	Improvement of FASD knowledge, dispenser group had higher scores (*p* < 0.01); lower PAC at follow-up, although PAC still prevalent (< 20%)	33/42 (8,15)^b^ or 78.6%
France et al. (2013) & France et al. (2014) Australia Randomised Controlled Trial Universal	4 groups; *n* = 354 non-pregnant women aged 18–45; inclusion criteria: alcohol consumption and *n* = 116 pregnant women (all in experimental condition, PAC and no reported PAC)	Message Framing Effects One threat only, one positive (self-efficacy) only and one threat and positive concept message group were compared with a control group, portrayed as a storyboard;	Persuasiveness of different message types, intention to/ confidence in ability to abstain or reduce alcohol consumption or PAC Single-item questions measured efficacy of message; Assessment of message factors through open- and closed-ended questions	All message framing types effective in reducing PAC; no significant difference between pregnant and non-pregnant women; messages including threat aroused negative emotions, self-efficacy only message aroused positive emotions	24/36 (3,13,14,15,16)^b^ or 66.7%
Ingersoll et al. (2013) USA Randomised Controlled Trial Primary, selective	3 groups (randomised); *n* = 73 women in EARLY condition group, *n* = 70 women (video group), *n* = 74 women (informational brochure group); mean age of 27.9 years; mean years of education = 13.6 years, 58.8% single, unemployed = 25.6%	Video, brochure, and motivational interviewing intervention (EARLY); single intervention	Comparison of outcomes (AEP risk reduction) to prior studies Baseline and follow-up test of AEP risk and alcohol consumption patterns, readiness to change assessed (contraceptive and drinking behaviour); 3- and 6-month follow-up; Comparison of data to two prior AEP studies (meta-analysis)	All interventions conditions effective in reducing AEP risk; EARLY condition had highest reduction rate in ineffective contraceptive behaviour and AEP risk; drinks per drinking day did not have a significant difference to other conditions; in comparison to other multi-session interventions, EARLY was not as effective	36/44 (15)^b^ or 81.8%
Ingersoll et al. (2018) USA Pilot Randomised Controlled Trial Primary, selective	2 groups (randomised); *n* = 36 women in CARRII and *n* = 35 women in static patient education group (control); inclusion criteria: AEPRG; mean age 27.8; 45% reported binge drinking	Web-based FASD intervention 2 groups, one group took part in an interactive and tailored web-based intervention (CARRII), other group utilised a static educational website intervention	Reduction of risky drinking, contraceptive behaviour and AEP risk Baseline, 9 weeks postintervention and 6-month follow-up assessment through online prospective diary	Individualized intervention (CARRII) leads to significant reduction in AEP risk; no significant change among static patient education group	40/44 (15)^b^ or 90.4%
Tenkku et al. (2011) USA Nonrandomized, Pre-Post Intervention Study Primary, selective	2 groups (self-selected); Total *n* = 458 (AEPRG, follow-up completion *n* = 319); web-based *n* = 373 at baseline, *n* = 260 at follow-up (completion rates from baseline 69.7%) mail-based *n* = 85 at baseline, *n* = 59 at follow-up (completion rates from baseline 70.2%)	Web- and mail-based FASD intervention Motivational messaging within four modules (self-guided change, individually tailored based on baseline answers)	Reduction of AEP risk (either alcohol consumption, contraceptive behaviour or both) Assessment of AEP risk (i.a. AUDIT), motivation and behaviour at baseline and 4-month follow-up	58% of participants no longer at AEP risk at follow-up (*p* < 0.001), no significant difference between groups, no demographic covariate significant; ¾ of women reduced or quit drinking	32/44 (15)^b^ or 72.7%
Wilton et al. (2013) USA Randomised Controlled Trial Primary, selective	2 groups; *n* = 68 women (telephone intervention) and *n* = 63 women (in-person) Inclusion criteria: AEPRG	Telephone or in-person intervention (2 sessions, standardised) Adapted from CHOICES and Healthy Mom’s Study; motivational interviewing and cognitive therapy techniques utilised; personalised feedback used (including goal setting and plan development)	Comparison of in-person and telephone administration of a brief AEP risk reduction intervention Questionnaire on demography, drug use, domestic violence, depression, eating disorders; TLFB methodology to determine alcohol use and sex (including contraceptive behaviour); after baseline and intervention, follow-up at 3, 6 and 12 months	No significant difference between in-person and telephone intervention; Small and significant reduction in alcohol use; large and significant increase in effective contraceptive behaviour; Significant reduction of AEP risk through effective use	35/40 (13,15,18)^b^ or 87.5%
Yu et al. (2010) USA Experimental Randomised Controlled Trial Universal	4 groups; *n* = 213 female students (18–25 years, m = 19.98; 2 pregnant participants); no inclusion criteria except for female gender	Message Framing Effects (consisting of loss vs. gain frames, statistic vs. exemplar appeals) 4 groups; each had to read 1 of 4 different FASD health messages	Evaluation of message framing effects and exemplification on FASD prevention intention (AEP risk reduction through fear of or behaviour/intention to prevent FASD) Post experimental questionnaire measuring affective responses, behavioural intentions, and attitudes	Both loss/ gain frames and statistics/exemplar appeals effective in increasing prevention intention; gain-statistics appeal promoted perceived efficacy; loss-exemplar appeal increased perceived severity and fear, prevention intention;	25/34 (3,13,14,15,16,18)^b^ or 73.5%

Table of Characteristics of included studies adapted from Crawford-Williams et al. (2015)

AEP = alcohol exposed pregnancy. AEPRG = alcohol exposed pregnancy risk group [non-pregnant, fertile, alcohol consumption, inefficient contraceptive behaviour, childbearing age (18–44)]. AUDIT = Alcohol Use Disorders Identification Test. BRFSS = Behavioural Risk Factor Surveillance System. CARRII = Contraception and Alcohol Risk Reduction Internet Intervention. CHOICES = Changing High-Risk Alcohol Use and Increasing Contraception Effectiveness Study. DFBI = Dual-Focused Brief Physician Intervention. EARLY = motivational interviewing and assessment feedback intervention by Ingersoll et al. (2013). FCL = Intervention Fidelity Checklists. HCP = healthcare professional. NVS = National Vital Statistics Natality Detail Files. PAC = prenatal alcohol consumption. PBD = prenatal binge drinking. SBD = single binge-drinking question. THCP = healthcare professionals from Treviso. TLFB = Timeline Followback. TPW = pregnant women from Treviso. VHCP = Healthcare professionals from Venetia. VPW = pregnant women from Venetia

Risky Drinking Behaviour (women) = consumption of an average of ≥ 8 standard drinks per week or engaging in binge drinking (i.e., ≥ 4 standard drinks in one day)

^a^Quality rated by using the quality rating system byMoncrieff et al. (2001)

^b^Criteria items excluded in quality assessment

Bazzo et al. (2015) used an observational comparative study design to evaluate an FASD health campaign that took place in Treviso by comparing healthcare professionals’ and pregnant women’s knowledge of and opinions about prenatal alcohol consumption to that of those in Verona, where the campaign did not take place. Additionally, they investigated the sources and kind of information that the groups either provided or received. The study showed that the campaign had long term positive effects on healthcare professionals’ knowledge and practice as they provided more information to their patients, although no significant difference was found among the pregnant women. The authors concluded that providing information alone is not an effective prevention strategy, as integrated and specialised approaches are needed. We identified the study’s limitations as being specific to population samples that limit the generalisation of the outcomes, the small sample size, and that the confounders were not controllable as it was purely an observational study.

Caley et al. (2010) evaluated the effectiveness of a workshop that focused on implementing FASD interventions using a case control study. Their sample consisted of health and human service professionals and took place four months after a workshop on FASD interventions. They found that 61% of the professionals initiated interventions, of which most were primary (59%). We found limitations in the low response rate (37%) and because no baseline was measured, no comparisons could be made prior to the workshop.

Cil (2017) evaluated the effectiveness of alcohol warning signs on decreasing prenatal alcohol consumption, prenatal binge drinking, and birth outcomes using a secondary data analysis by comparing data from national natality statistics and national surveys on behavioural risk taking. They found that AWS laws led to an 11% decrease in prenatal alcohol consumption odds and a 75% decrease in prenatal binge drinking odds. Furthermore, a change in prenatal alcohol consumption was largest amongst primipara and women who were over 30. Significant differences after the implementation of AWS laws in FASD birth outcomes or incidence rates could not be found. Cil theorised that the lack of a significant correlation between AWS laws and FASD birth outcomes was due to lack of diagnoses at birth. We found that limitations of the study included the lack of comparability of the data, as the author compared samples with different sources and years of origins. Additionally, the apperception of AWS was not measured, i.e., whether or not individuals were even aware of AWS, so evidence for a direct link between AWS and lower prenatal alcohol consumption was lacking, as was the role that other factors may have played in the observed decreases.

France et al., (2013, 2014) evaluated the efficacy of different message types regarding their persuasiveness, including their influence on the intention to and confidence in a person’s ability to abstain from or reduce alcohol consumption or prenatal alcohol consumption. Results of this RCT showed that there was a general significant increase in intention and confidence in abstaining or reducing alcohol consumption, although threat concept messages were the most effective in increasing behavioural intentions and confidence in possible modification. The authors concluded that threat messages should be implemented in preventive messages and campaigns, while adding that self-efficacy concepts in communication helped decrease potential reactions, emotions, and cognitions. Thus, a combination of concepts was found to be the best way to develop preventive messages. Limitations of the study included difficulties in generalizing from the findings, as women with a lower socio-economic status were not included in the study.

In a pilot RCT, Ingersoll et al. (2018) evaluated the efficacy of an automated, individually tailored intervention on AEP risk versus a static educational website. The study focused on risky drinking and contraceptive behaviour. Significant outcomes included reductions in unprotected sex, risky drinking, and AEP risk amongst the specialised intervention website group targeted. There was no significant change amongst the static educational website group. The authors concluded that if the participants utilised the program more, they were more likely to have experienced a change in their behaviour. Additionally, participants were more likely to finish all core modules in the individualised website. A limitation of the study was that the statistical power was weak and that larger sample sizes would be needed to further validate the findings.

Using an RCT, Yu et al. (2010) evaluated the effect of different message framing types (statistic vs. exemplar appeals, loss vs. gain appeals) on the intention to prevent FASD. The messages were designed to look like public service announcements found in newspapers. Results showed that exemplar appeals that were loss-framed elicited significant levels of fear, whereas gain-framed messages increased efficacy. Yu et al. (2010) concluded that each message frame had advantages and that message goals should be considered when implementing or creating awareness campaigns utilising framing effects. The authors concluded that a limitation of the study was the selective sample, as the students did not view themselves as at risk of pregnancy.

Tenkku et al. (2011) evaluated the effectiveness of a web-based intervention in reducing AEP risk using a nonrandomized, pre-post intervention study. The sample consists of AEP at-risk women who self-selected into two delivery methods, either web- or mail-based. The interventions consisted of four modules based on motivational messaging that were specifically tailored to the individuals’ answers and needs. The study had a follow-up of four months after baseline. Outcomes showed a significant decrease in AEP risk, although no significant difference could be found between the groups. The authors concluded that a self-guided intervention utilising motivational interviewing techniques showed effectiveness in preventing AEP. We determined that limitations of the study included the self-reports, low follow-up rates, and insufficient, unbalanced sample groups.

Ingersoll et al. (2013) used an RCT to evaluate the efficacy of a one-session motivational AEP prevention intervention. For this, women were assigned to three groups, the first of which consisted of an individual, face-to-face, 60-min single counselling session that utilised motivational interviewing (EARLY). Participants in the second group watched an informational video, received a 5-min briefing from a counsellor, and were offered brochures. The third group only received informational brochures. Results showed that all intervention conditions effectively reduced AEP risk, and that the individualised face-to-face condition showed the highest reduction rates in ineffective contraceptive behaviour and AEP risk. The brochure condition proved to be more efficient than the video condition. The authors theorised that this could be due to possible stereotyping and stigmatisation that resulted from the contents of the video, as well as differences in general contents between the two conditions. This could explain why the motivation to change was lower, as the women in that condition group did not view their behaviour as extreme as that of the women portrayed in the video. The authors suggested a lack of non-TLFB control group and possible cross-contamination due to the same counsellors implementing all conditions, as limitations to their study.

Using an RCT, Wilton et al. (2013) compared two sessions of an in-person and telephone administration of a brief AEP risk reduction intervention. Results showed a significant but small reduction in alcohol use, as well as a large and significant increase in effective contraceptive behaviour. In general, the intervention resulted in a significant reduction in AEP risk and differences between administration types were not found. The authors concluded that brief telephone interventions could be used successfully and were more cost-effective than in-person interventions. Additionally, the study showed that it might be easier to increase effective contraceptive use, as women of childbearing age might not be interested in decreasing their alcohol consumption due to their current lifestyle. The authors suggested the small sample size, funding limitations that lead to change in counsellors in follow-up with possible rapport differences, and possible recollection errors in the TLFB due to the nature of self-reports, as limitations to their study.

Driscoll et al. (2018) evaluated the effectiveness, acceptability, and feasibility of FASD prevention messages in women’s restrooms in a formative evaluation study. The authors placed health communication messages on pregnancy test dispensers or as posters in women’s restrooms in establishments that served alcohol. These health messages included information on outcomes of FASD, as well as numbers for telephone carelines while addressing contraceptive behaviour. Pregnancy tests and condoms were distributed for free or for a small charge in the establishments. Results showed that pregnancy tests that were free were more likely to have been used. Both health message distribution types were effective in increasing knowledge, but the dispensary type proved the most effective. Although prenatal alcohol consumption was lower at follow-up, it was still prevalent (< 20%). We found limitations of the study in the lack of baseline data collection and control groups. Additionally, it was unclear exactly how participants were recruited for baseline; the only information given was that recruitment took place in the communities in which the messages were distributed.

## Discussion

We found 10 studies with different approaches and target groups that measured a variety of outcomes related to FASD including awareness, knowledge, self-efficacy, and behaviour change. In general, the approaches used in the studies reviewed can be effective in preventing FASD. Universal approaches like alcohol warning signs and labels on alcoholic products seemed to be effective in decreasing prenatal alcohol consumption odds, although they had only a limited impact on FASD incidence rates (Cil, 2017). However, inadequate diagnostic practices might also play a role in prevalence and incidence rates, including miscarriages or stillbirths due to prenatal alcohol consumption, which was not included in FASD incidence.

This systematic review showed that there is a lack of research concerning universal and primary intervention or prevention strategies targeting FASD. Additionally, the majority of the studies included demonstrated methodological shortcomings or even deficits, even if they demonstrated significant effects. Only five of the ten studies used RCTs, and small sample sizes hindered the generalisation of their results. Furthermore, many studies were not as detailed or transparent as required to understand their contents and procedures, like that of Caley et al. (2010), which limited the study quality rating. In contrast, researchers such as Driscoll et al. (2018), Cil (2017), and Wilton et al. (2013), as well as both studies by Ingersoll et al., (2013, 2018), demonstrated exemplary transparency and highly detailed summaries of their work. Additionally, it must be noted that another limitation of the studies was that few high-risk women were included as participants and the authors believed that FASD preventive activities were in principle only effective if they also reached these high-risk groups.

On the basis of the high quality studies included, we can make five observations. The more individualised and tailored intervention programs were, the higher the reduction in AEP risk seemed to be (Ingersoll et al., 2018); structural preventions alone did not seem to be sufficient to reduce FASD incidence (Cil, 2017); even very brief interventions seemed to be effective in reducing AEP risk; and motivational interviewing techniques seemed to show better results in decreasing risky behaviour than other techniques did (Ingersoll et al., 2013). Further, it seemed to be easier and more fruitful to increase effective contraceptive behaviour than to decrease alcohol use (Wilton et al., 2013), and raising awareness in environments where alcohol consumption was prevalent seemed to decrease prenatal alcohol consumption and increase FASD knowledge (Driscoll et al., 2018).

Motivational interviewing techniques have shown promising signs in changing behaviour in indicated or at-risk groups (Ingersoll et al., 2013; Tenkku et al., 2011; Wilton et al., 2013). This may also be due to an increase in self-efficacy. Health campaign messages that are not aimed at specific risk groups need to determine what goal they have related to FASD prevention. In general, it seems to be useful to mix message framing and appeals to increase persuasiveness, as a balance of negative and positive emotions and the responses they elicit in each individual decrease defense mechanisms (France et al., 2013, 2014).

Although follow-ups were implemented in the studies reviewed, the only one that examined the prolonged cognitive and behavioural outcomes of health campaigns and interventions was by Bazzo et al. (2015). While it showed significant effects amongst health care professionals, the general population seemed to require more constant awareness- raising interventions. It could be concluded that an effective intervention to decrease AEP risk and FASD prevalence would have been to administer a continuous educational campaign amongst all relevant ages. These educational campaigns should be easily accessible and could be delivered through either web- or mail-based platforms or via public advertisements. They should include offers for counselling and advice free of charge as to be accessible to people of all economic backgrounds. According to Driscoll et al. (2018), people were more likely to implement intervention tools such as pregnancy tests and condoms if these were free. Such interventions should be evaluated using longer observational periods, like in the study by Bazzo et al. (2015).

Concerning public awareness and contraceptive behaviour, a universal population approach has the additional benefit of including men. While effective contraceptive behaviour requires men’s participation, previous studies have proven that the social environment (i.e., partners including sexual partners) also influences AEP risk and prenatal alcohol consumption (Clarke & Gibbard, 2003; Gupta et al., 2016; Landgraf & Heinen, 2016a). The authors suggested that a women’s social environment can address problematic AEP risk behaviour and have a positive impact, with better FASD knowledge and awareness by individuals within the women’s social environment further decreasing the risk.

Studies that focused on healthcare professionals demonstrated that they require further training to increase intervention practices and knowledge related to FASD and its diagnosis. Nevertheless, these trainings were found to be effective and showed long-term effects (Balachova et al., 2013). With respect to educating healthcare professionals, findings reported by Bazzo et al. (2015) illustrated the importance of FASD training courses. Other studies have also found a general lack of knowledge and awareness of FASD and its diagnosis as well as confidence in recognising and treating FASD amongst healthcare professionals (Mukherjee et al., 2015; Payne et al., 2011).

We conclude that future studies on FASD prevention should have greater sample sizes, include long-term follow-ups to assess the actual efficacy of the interventions, conduct large-scale cohort studies and also consider including men as participants. Additionally, researchers should consider focussing on integrating intervention activities further, including school-based activities and campaigns, to reach as many potential risk groups as possible. Furthermore, they should consider studying the efficacy of integrating FASD prevention into general messages targeting health promotion, like connecting FASD awareness brochures or similar information guides to HPV (human papillomavirus) vaccinations to reach a younger audience.

When comparing the number of publications and studies on specific risk groups based on factors such as ethnicity or socioeconomic status, universal primary prevention appears to be insufficiently studied. The studies reviewed emphasized the effectiveness of educational and awareness programs in raising FASD knowledge. However, it should be noted that studies that only measure knowledge and not actual behavioural changes have limited utility in measuring the likelihood that they may actually change behaviour.

Additionally, many of the studies reviewed were conducted in the USA. This raises the question of whether their results can be fully generalised amongst other parts of the world, such as Europe and developing countries, due to cultural and educational differences as well as other prevalence rates. There is still much that needs to be done.

## Conclusions

FASD is a highly prevalent serious neurodevelopmental disorder that is preventable. The studies we reviewed showed that different types of approaches can be effective in raising FASD knowledge and therefore potentially changing behaviour. However, more high-quality studies are needed to build a robust evidentiary base as to the efficacy of FASD prevention. That said, the studies included in this systematic review can serve as a guide for developing such FASD prevention approaches.
